# Study on Newly Isolated *Dysmorphococcus* Strains from Reunion Island as Potential Sources of High-Value Carotenoids

**DOI:** 10.3390/foods13233922

**Published:** 2024-12-04

**Authors:** Samuel Jannel, Yanis Caro, Marc Bermudes, Thomas Petit

**Affiliations:** 1 Laboratoire de Chimie et de Biotechnologie des Produits Naturels, ChemBioPro (EA2212), Université de la Réunion, 15 Avenue René Cassin, FR-97490 Sainte-Clotilde, La Réunion, France; 2 Green Mascareignes Technologies SAS, 2 rue Maxime Rivière, FR-97490 Sainte-Clotilde, La Réunion, France; 3 Département HSE, IUT de La Réunion, 40 Avenue de Soweto, FR-97410 Saint-Pierre, La Réunion, France

**Keywords:** *Dysmorphococcus*, astaxanthin, canthaxanthin, biodiversity, reunion island, photobioreactor, new application

## Abstract

Certain secondary carotenoids, such as astaxanthin and canthaxanthin, are of growing economic interest in the fields of human nutrition, food, health and cosmetics, as well as feed and aquaculture, particularly due to their numerous biological activities, such as their remarkable antioxidant properties. The present study was devoted to assessing, in a photobioreactor, the feasibility of cultivating newly isolated *Dysmorphococcus* strains from the biodiversity of Reunion Island for the production of these valuable xanthophylls. The results showed that all these strains were capable of producing and accumulating canthaxanthin and astaxanthin in response to environmental stresses. Among them, a strain which presented interesting morphological, genetic and biochemical properties as compared to the other *Dysmorphococcus* strains was further cultivated in a 3 L benchtop photobioreactor and was found to produce maximum carotenoid-rich biomass concentrations and productivities of about 4 g L^−1^ dw and 0.055 g L^−1^ d^−1^ dw, respectively. We also found that the biomass contained up to 1.2 mg g^−1^ dw of canthaxanthin and 0.7 mg g^−1^ dw of different forms of astaxanthin, mainly astaxanthin monoesters. The productivity of these carotenoids was found to be lower than those observed for other microalgal species previously reported, and we suggested that further optimizations with respect to the cultivation and the carotenogenesis induction processes are needed to improve productivities and to make this locally isolated *Dysmorphococcus* strain useful for future commercial production of natural canthaxanthin and astaxanthin.

## 1. Introduction

Carotenoids are tetraterpenoid pigments that can be produced naturally by plants, algae, some bacteria and fungi. Due to their diverse bioactive and chemical properties, many of them present a growing economic interest in the fields of food, nutrition, health and cosmetics as well as feed and aquaculture [1]. In particular, astaxanthin, mostly valued for human applications as a dietary supplement, and canthaxanthin, which can be used as a food additive [2], but also β,β-carotene, lutein, lycopene and zeaxanthin, are among the most valuable carotenoids found in these markets [1,3]. Some economic analysts estimated that the global market of carotenoids will reach around USD 2.5 billion in 2024 and should grow up to USD 3.4 billion in 2029 with a compound annual growth rate of more than 6% (marketsandmarkets.com; accessed on 1 October 2024) [3].

*Dysmorphococcus* is a genus of cosmopolitan phytoplanktonic freshwater green microalgae which belongs to the family Phacotaceae, the order Chlamydomonadales in the class Chlorophyceae. This genus is composed of unicellular, uninucleate and biflagellate microalgae, with flagella anterior of equal length, whose protoplast is contained in a rigid lorica [4]. As is the case for the family to which it belongs, it was suggested that *Dysmorphococcus* is a polyphyletic genus because morphological and structural characteristics vary extensively between the different species [5]. Species differentiation is partly based on the size and the shape of the protoplast and of the lorica, the number of pyrenoids and contractile vacuoles, the shapes of the papilla if present and of the stigma, and the nature of the lorica ornamentation [4,6]. To date, 12 different species whose names are accepted taxonomically are referenced in the Algaebase database [4], and the holotype species of this genus is *D. variabilis* H. Takeda [7]. Few studies are devoted to this genus and most of them address its taxonomy, phylogeny, morphology and physiology [6,8,9,10,11]. Among the different species, *D. globosus* Bold & Starr [12] is certainly the most documented [5,13] and the only identified species whose genetic sequences have been deposited in the GenBank database. For instance, it is the only *Dysmorphococcus* species whose potential economic interest has been reported since the filing of a patent relating to the lipolytic activity of an extract which could be used as an active ingredient in cosmetic or pharmaceutical compositions [14]. More recently, it was claimed that a new strain isolated from the Himalayas was able to produce and accumulate astaxanthin with higher productivity than the commercially used microalgal species *Haematococcus lacustris* [15].

In a previous study, eight microalgal strains were monoclonally isolated from the freshwater biodiversity of Reunion Island and were morphologically and genetically identified as very closely related to the genus *Dysmorphococcus* [16]. This study showed a relative genetic variability between these strains themselves but also with *D. globosus*, allowing one to assume that it could be another species. Moreover, preliminary chromatographic analyses of pigment extracts from some of them confirmed their ability to produce and accumulate secondary carotenoids such as astaxanthin in response to environmental stresses such as high levels of light and/or nutrient deprivation.

Reunion Island is an overseas French island located in the Indian Ocean between Madagascar and Mauritius. This territory, recognized as one of the 34 global biodiversity hotspots worldwide [17], presents a unique biodiversity due to the variety of climatic conditions generated by its specific mountainous and volcanic relief. In order to reduce its economical and material dependencies due to its insularity, while meeting the expectations for external markets, the territory has expressed its ambition to promote the “green economy”, notably through the valorization of natural products from terrestrial and marine biodiversity in domains such as food, nutrition, health and cosmetics [18]. In this context, highlighting new local sources of valuable carotenoids could represent a real opportunity to contribute to achieving this objective.

The present study is devoted to the further characterization, using chromatographic and spectrometric techniques, of secondary carotenoids produced by the previously isolated local *Dysmorphococcus* strains in response to environmental stress conditions. These analyses aimed to highlight the ability of these strains to produce carotenoids of potential economic interest in the fields of nutrition and food, as well as to compare carotenoid profiles in order to assess any differences that might reflect genetic variabilities between the strains. In addition, the study presents preliminary culture scale-up tests for two of these strains from 250 mL flasks to a 3 L benchtop photobioreactor. These tests gave us an initial estimate of the potential biomass and carotenoid productivities that could be obtained for a strain selected for its genetic and biochemical particularities. Overall, the results should allow us to assess the opportunity to cultivate these *Dysmorphococcus* strains at a larger scale to produce high-value secondary carotenoids and whether they could represent a valuable alternative to *H. lacustris* for astaxanthin production.

## 2. Materials and Methods

### 2.1. Chemicals and Reagents

Nutritive media composed of mineral components purchased from Carlo Erba Reagents (Val-de-Reuil, France) as well as dichloromethane, methanol, acetone and methyl tert-butyl ether (MTBE), which were of analytical grade. All-*trans*-astaxanthin (purity ≥ 97%) and all-*trans*-canthaxanthin (purity ≥ 95%) analytical standards as well as 2-Tert-butyl hydroquinone (TBHQ) (purity ≥ 97%), HPLC-grade MTBE and ammonium acetate were obtained from Merck (Darmstadt, Germany). β, β-carotene (purity ≥ 95%), cryptoxanthin (purity ≥ 95%), lutein (purity ≥ 92%) and zeaxanthin (purity ≥ 95%) standards were supplied by Extrasynthese (Genay, France), while the reference extract of astaxanthin esters from *H. lacustris* was purchased from Cluzeau Info Labo (Sainte-Foy-la-Grande, France).

### 2.2. Microalgal Strains

**Figure 1:**
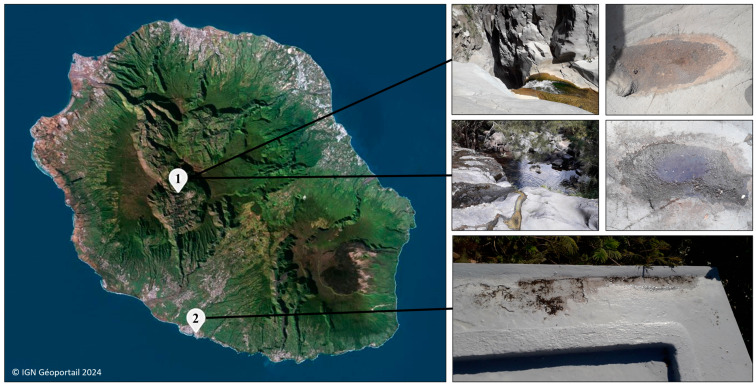
A map of Reunion Island; photographs of sample collection locations concerning the *Dysmorphococcus* strains isolated during our previous study: (1) Bras-Rouge river, Cilaos, for the strains’ CBRs; (2) Terre-Sainte, Saint-Pierre, for the strain M8.

*Dysmorphococcus* strains were monoclonally isolated from freshwater samples collected in different locations across Reunion Island during our previous study [16]. The *Dysmorphococcus* strains’ CBRs were isolated from samples collected on 3 April 2019 near the Bras-Rouge river in the cirque of Cilaos at an approximate altitude of 900 m (coordinates: 21°07′44″ S, 55°27′19″ E). Among these strains, CBR1, CBR2 and CBR5 came from a sample which consisted of orange crusts scraped from a dried puddle on a rock while CBR3, CBR4, CBR7 and CBR11 came from water and sediments collected from a puddle. The *Dysmorphococcus* strain M8 came from crusts scraped from a puddle on a rooftop in Terre-Sainte, Saint-Pierre, on 17 June 2021, at an approximate altitude of 30 m (coordinates: 21°20′34″ S, 55°28′57″ E) (Figure 1). The sampling campaign was carried out within the framework of a sampling authorization granted by the Reunion Island National Park (Decree No. DIR-I-2024-179). After collection, samples were stored in isothermal containers during transport to the laboratory, where strains were monoclonally isolated and cultured in vitro, as was described in our previous study [16].

**Figure 2:**
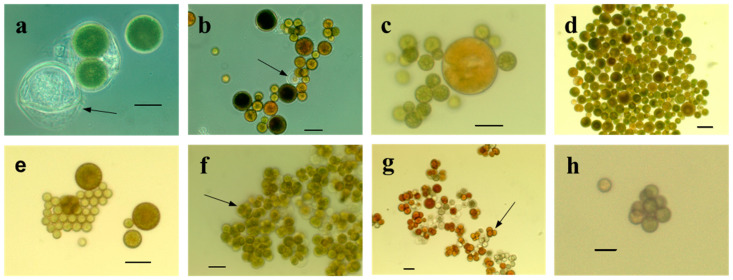
Microphotographs of some monoclonally isolated *Dysmorphococcus* strains: (**a**) CBR1; (**b**) CBR2; (**c**) CBR5; (**d**) CBR3; (**e**) CBR4; (**f**,**g**) CBR11; (**h**) M8. (**a**,**b**) Phase contrast; (**c**–**h**) bright field. (**a**,**c**,**e**–**h**) Scale bar 25 µm; (**b**,**d**) scale bar 50 µm. (**a**,**b**) Arrows show parental cell walls after the release of daughter cells; (**f**,**g**) arrows show cell clusters.

In the previous study, after morphological characterization (Figure 2), molecular and phylogenetic analyses on amplified sequences of the 18S rRNA and *rbc*L genes allowed us to suggest a link between these strains and the *Dysmorphococcus* genus and highlighted genetic variabilities between the isolated strains themselves as well as with *D. globosus* [16].

### 2.3. Algal Cultivation, Carotenogenesis Induction and Biomass Preparation

All isolated *Dysmorphococcus* strains were previously grown in a sterile and monoclonal manner in batch mode with increasing volumes of Bold’s Basal liquid nutrient medium (BBM) [19] ranging from 25 mL culture flasks to 250 mL glass flasks. These cultures were incubated in a climate chamber (Pol-Eko, Wodzisław Śląski, Poland) equipped with white LED tubes at 20 °C and under moderate irradiation (40–50 µmol photons m^−2^ s^−1^) to avoid inducing highlight stress and photo-inhibition and with a 14:10 h light/dark cycle. Secondary carotenoid production was induced by nutrient deprivation. Next, 2 L Duran bioreactors (Schott AG, Mainz, Germany) containing BBM with triple nitrogen concentrations (3N-BBM) were inoculated with part of the 250 mL flask cultures of strains CBR11 and CBR1 and incubated at room temperature, under illumination composed of 100% white LED light at moderate irradiances (90 µmol photon m^−2^ s^−1^) and with a 12:12 h light/dark cycle. Agitation was provided by a magnetic stirrer and air bubbling. Secondary carotenoid production was induced by nutrient deprivation and illumination with higher irradiance (>200 µmol photons m^−2^ s^−1^). Cultures grown in 2 L bioreactors were partially used as inoculums to grow CBR11 and CBR1 strains in 3 L FMT 150/3000 benchtop photobioreactors (Photon Systems Instruments (PSI), Drásov, Czech Republic), which consisted of a thermoregulated vertical glass parallelepiped tank, illuminated on one side by an LED panel and equipped with a vertical magnetic stirrer. Cultures were carried out in batch mode, using 3N-BBM, at a temperature maintained at 25 °C and under continuous illumination consisting of 100% white LED light. The different light regimes applied involved gradually increasing irradiance in the range 20 to 600 µmol photons m^−2^ s^−1^ depending on cell densities to ensure sufficient light access to the cells while avoiding inducing highlight stress and photo-inhibition during vegetative growth. On the contrary, when it was observed that nutrient deprivation linked to non-renewal of the culture medium began to induce carotenogenesis, irradiation was increased in the range 750 to 1500 µmol photons m^−2^ s^−1^ to generate highlight stress, which enhances carotenogenesis in combination with nutritional stress. In these cultures, pH was maintained at around 7.5 to 8.5 by the occasional addition of 1N HCl solution, and agitation was provided by a magnetic stirrer and air bubbling. Punctually, 100 mL of the cell suspensions was harvested by centrifugation in pre-weighed 50 mL conical tubes at 3000× *g* for 10 min at room temperature. The pellets were rinsed twice with distilled water and stored at −80 °C for subsequent lyophilization using a FreeZone 2.5 lyophilizer (Labconco, Kansas City, MO, USA). The freeze-dried biomasses were then weighed using an analytical balance and stored for further analysis. When microscopic observations showed that almost all cells were orange, whole cultures from 3 L photobioreactors were harvested and processed as described above.

### 2.4. Growth Analyses

For growth analyses, 1 mL samples of each culture were regularly collected and fixed with Lugol’s solution (at 2% in the samples) in order to measure the growth in the vegetative stage. The cell densities (Cds) were estimated by counts on four replicates of the same samples under light microscope (MOTIC BA310, Xiamen, China) using a Malassez hemocytometer (Marienfeld Superior, Lauda-Königshofen, Germany) and were expressed as number of cells per mL. Based on these counts, the growth curves representing the cell densities in the function of the cultivation time were plotted and enabled the calculation of the following parameters: the maximum specific growth rate (*µ_max_*), expressed as day^−1^, representing the number of cell divisions per day during the exponential phase of the growth curve, and the minimum doubling time (*td_min_*), expressed as day, corresponding to the time required for doubling the cell density during the exponential phase:

$$\mu_{max}=\frac{\ln\left(N2/N1\right)}{\left(t2-t1\right)}$$

$${td}_{min}=\frac{ln2}{\mu_{max}}$$

where *N*2 and *N*1 were the cell densities at *t*2 and *t*1, respectively, which corresponds to days in exponential phase. Moreover, to have a more global view of the growth, the mean growth rates (*µ_mean_*) and the mean doubling times (*td_mean_*) were also calculated on the basis of longer durations of the growth phase.

### 2.5. Carotenoid Extraction

The freeze-dried biomasses (10 mg) were homogenized using a 15 mL Dounce homogenizer and pigments were extracted with 2 mL of a mixture of dichloromethane/methanol (25:75, *v*/*v*) in darkness, at ambient temperature and under a nitrogen atmosphere [20]. The mixture was then centrifuged at 10,000× *g* for 5 min and the supernatant was collected. The extraction procedure was repeated until the pellet was colorless (at least 5 times), and supernatants were combined and evaporated to obtain a volume of 10 mL (adapted from [20]). The obtained pigment extracts were stored at −20 °C for subsequent saponification and/or high-performance thin layer chromatography (HPTLC), high-performance liquid chromatography (HPLC) and mass spectrometry analyses. One volume of 0.1 M NaOH in methanol for four volumes of pigment extract was added to saponify xanthophyll esters on some extracts, and these mixtures were kept overnight at 5 °C under nitrogen in darkness until complete saponification (adapted from [21]).

### 2.6. Carotenoid Extract Analyses

Qualitative analyses were performed using a HPTLC Camag (Muttenz, Switzerland) in reverse phase on 20 × 10 cm glass-packed C18 RP HPTLC silica gel plates with 0.2 mm layer thickness (Merck, Darmstadt, Germany). The system was composed of an Automatic TLC Sampler 4, an Automatic Developing Chamber, a TLC Visualizer 2 and a TLC Scanner 4. Prior to use, the plates were pre-developed in a mixture of methanol/dichloromethane (1:1, *v*/*v*) and dried for 20 min at 100 °C in an oven. In volumetric flasks (Simax, Sázava, Czech Republic), carotenoid standards (1 mg free astaxanthin, 5 mg lutein, 5 mg zeaxanthin, 1 mg cryptoxanthin, 10 mg β,β-carotene and 2 mg canthaxanthin) were individually dissolved in acetone to obtain stock solutions at concentrations of 20 µg mL^−1^. From these stock solutions, a carotenoid standard mix solution was prepared by mixing 200 µL of each one into a 1.5 mL vial. The standard mix solution and the samples were applied with a volume of 50 µL. Moreover, a reference extract of astaxanthin esters from *H. lacustris* (100 mg) was also dissolved in acetone to prepare a pigment solution at 100 µg mL^−1^ which was applied to a volume of 20 µL. Then, the plates were developed in a mixture of methanol/acetone (1:1, *v*/*v*) containing 0.1% TBHQ [22,23]. Finally, the developed plates were visualized under white light and UV at 366 nm to assess the profile of all pigments and chlorophylls, respectively, and scanned at wavelengths between 190 and 900 nm.

Qualitative and quantitative analyses were conducted in reverse-phase HPLC using a Vanquish UHPLC System (Thermo Fisher Scientific, Waltham, MA, USA) equipped with a photodiode-array detector. Separation of the pigments was performed on a carotenoid C_30_ column (150 × 4.6 mm i.d., 3 µm) (YMC, Kyoto, Japan) connected to a guard cartridge (10 × 4.0 mm i.d., 3 µm) at 25 °C. The mobile phase consisted of methanol (A) and methyl tert-butyl ether (MTBE) (B) and the following gradient was applied: 0–8 min, 0% B; 8–14 min, 0–22% B; 14–24 min, 22% B; 24–29 min, 22–40% B; 29–45 min, 40% B. The flow rate was 1.0 mL min^−1^ and the injection volume was 20 µL. The chromatographic peaks were measured at a wavelength of 480 nm and spectra were acquired in the range from 200 to 800 nm in a 3D field mode (adapted from [24]). The dosage of canthaxanthin and astaxanthin in the extracts was performed against calibration curves plotted from the analyses of analytical standard solutions of each carotenoid at concentrations of 5, 10, 20, 30 and 40 µg mL^−1^ prepared in acetone using volumetric flasks.

Liquid chromatography coupled with mass spectrometry (LC-MS) was performed using an UPLC-DAD-MS Shimatdzu (Kyoto, Japan) using the same method as for the HPLC analyses except in positive ionization mode, for which eluent A consisted of methanol with 28 mM of ammonium acetate. The electrospray ion (ESI) source was operated in positive and negative ionization modes with capillary voltages of 4.5 kV and −4.5 kV, respectively. Mass spectra were scanned in the mass range (*m*/*z*) of 100 and 900.

### 2.7. Statistical Analyses

Cellular densities were presented as mean ± standard deviation. They were estimated with the count of four replicates of the same sample. The outliers were identified using the Dixon and the Grubbs tests, with a significance level α of 0.05, and replaced by the sample’s average. Then, data were submitted to a one-way analysis of variance (ANOVA) to measure the statistical differences among the means. In this analysis, the normality and the homoscedasticity were analyzed using the Shapiro–Wilk and the Levine tests, respectively (α = 0.05), while Tukey’s post hoc test was used to compare differences which were considered statistically significant when the *p*-value < 0.05. All statistical analyses were performed using the XLSTAT software version 2023.3.1 (XLSTAT statistical and data analysis solution was purchased from Addinsoft and accessed from https://www.xlstat.com/fr on 12 March 2024).

## 3. Results

**Figure 3:**
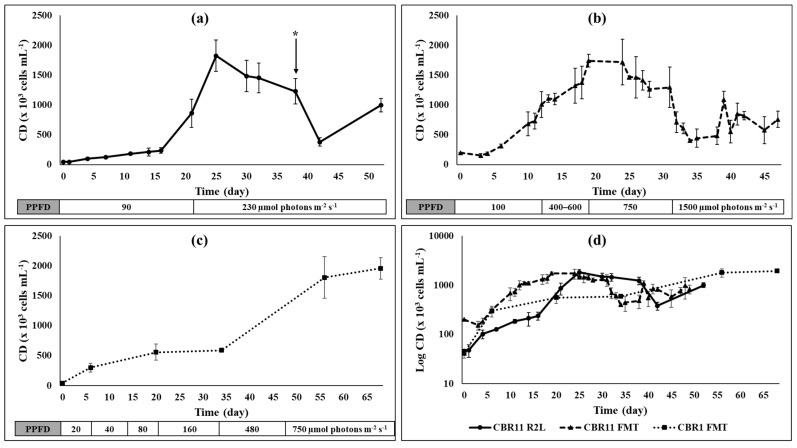
Growth kinetics of the *Dysmorphococcus* strains CBR11 and CBR1 cultivated in batch mode with the 3N-BBM medium under increasing photosynthetically active photon flux density (PPFD): (**a**) CBR11 in a 2 L glass reactor; (**b**) CBR11 in a 3 L benchtop photobioreactor; (**c**) CBR1 in a 3 L benchtop photobioreactor; (**d**) comparison between the three cultures where cell densities are expressed in a logarithmic scale. Bars represent standard deviations on 4 replicates of the same sample; arrow with (*) represents a harvest of 500 mL of culture replaced by fresh medium.

**Figure 4:**
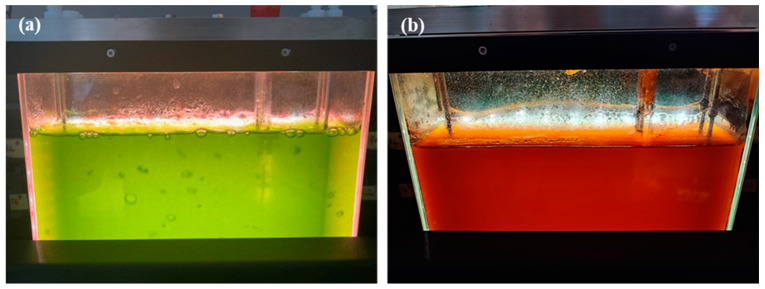
Photographs of the culture of the *Dysmorphococcus* strain CBR11 cultivated in a 3 L benchtop photobioreactor with the 3N-BBM medium: (**a**) after 13 days; (**b**) after 77 days.

**Figure 5:**
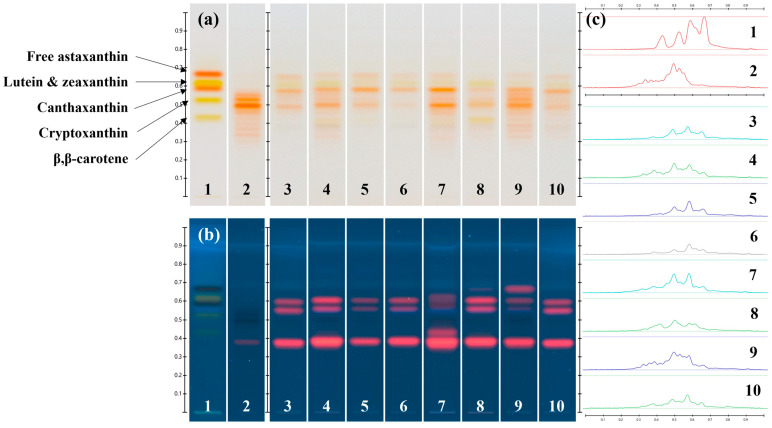
HPTLC chromatograms of pigment extracts obtained from the isolated *Dysmorphococcus* strains: (**a**) under transmitted white light illumination; (**b**) under reflected 366 nm light illumination; (**c**) densitogram for the transmitted white light illumination. Tracks 1: carotenoid standards; 2: astaxanthin esters from *H. lacustris* reference extract; 3: CBR1; 4: CBR2; 5: CBR3; 6: CBR4; 7: CBR5; 8: CBR7; 9: CBR11; 10: M8.

**Figure 6:**
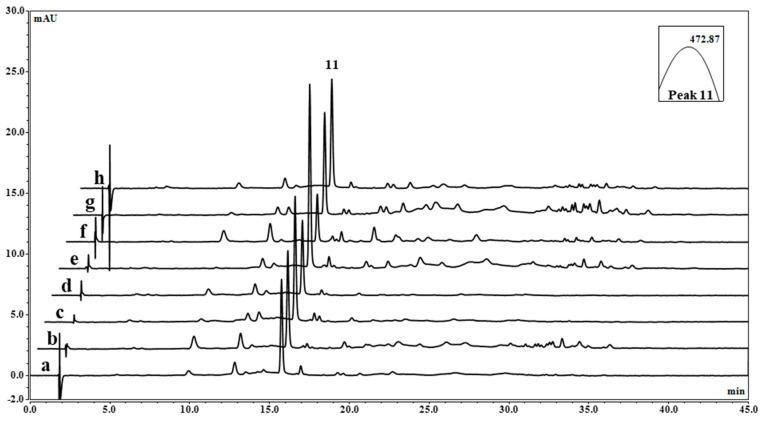
HPLC chromatograms of pigment extracts obtained from the isolated *Dysmorphococcus* strains: a: CBR1; b: CBR2; c: CBR3; d: CBR4; e: CBR5; f: CBR7; g: CBR11; h: M8. Peak 11 corresponded to that of Table 2. Detection with DAD at 480 nm.

**Figure 7:**
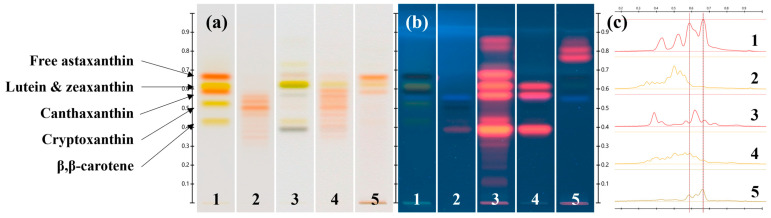
HPTLC chromatograms of pigment extracts obtained from the isolated *Dysmorphococcus* strain CBR11: (**a**) under transmitted white light illumination; (**b**) under reflected 366 nm light illumination; (**c**) densitogram for the transmitted white light illumination. Track 1: carotenoid standards; track 2: astaxanthin esters from *H. lacustris* reference extract; track 3: during the vegetative green stage; tracks 4 and 5: during the aplanospore orange stage before and after saponification, respectively. The lines on the densitogram indicate R_F_ of canthaxanthin (left) and free astaxanthin (right).

**Figure 8:**
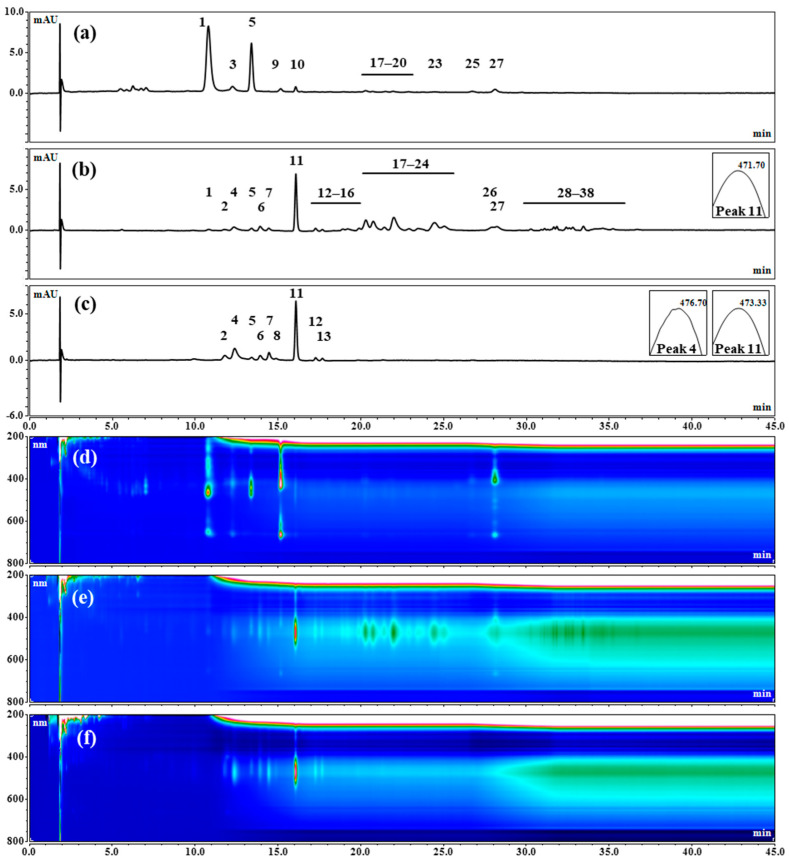
HPLC chromatograms of pigment extracts obtained from the *Dysmorphococcus* strain CBR11: (**a**,**d**) in the vegetative green stage; (**b**,**e**) in the aplanospore orange stage; (**c**,**f**) in the aplanospore orange stage after saponification; (**a**–**c**) detection with DAD at 480 nm; (**d**–**f**) detection in a 3D field mode.

**Figure 9:**
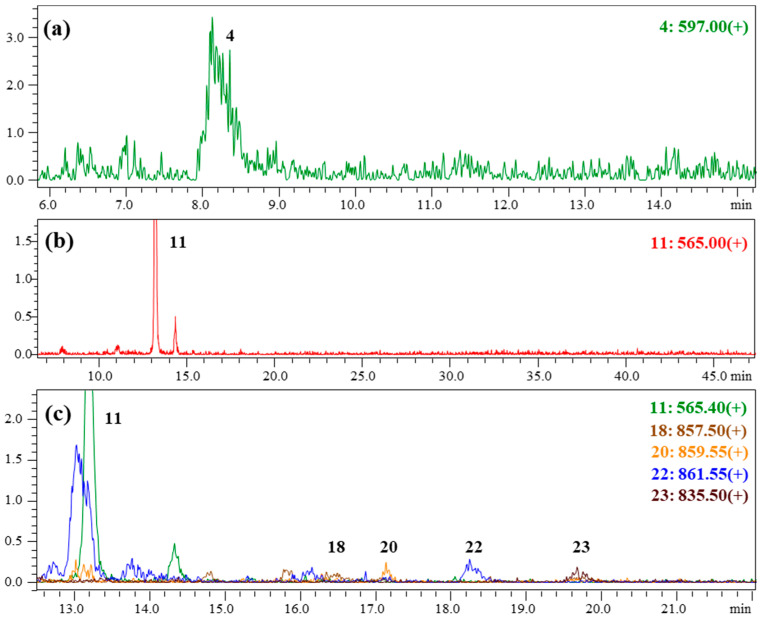
LC-MS chromatograms of the extract obtained from the orange biomass of the isolated *Dysmorphococcus* strain CBR11: (**a**) free astaxanthin; (**b**) canthaxanthin; (**c**) astaxanthin monoester. The ratios *m*/*z* of the positive ions [M + H]^+^ corresponding to each peak are indicated to the right of the chromatograms.

The results obtained from the preliminary cultures at a benchtop photobioreactor scale showed that the isolated *Dysmorphococcus* strains CBR1 and CBR11 could grow satisfactorily in batch mode in the 3N-BBM medium under increasing photosynthetically active photon flux density (PPFD) from 20 to 600 µmol photons m^−2^ s^−1^ (Figure 3). Indeed, the cultures of these two strains in a 3 L photobioreactor presented maximal specific growth rates (*µ_max_*) of around 0.32–0.33 d^−1^, maximal cell densities reaching 1800–2000 × 10^3^ cells mL^−1^ and biomass concentrations between around 4 and 5.5 g L^−1^ in dry weight (dw) corresponding to biomass productivities from 0.055 to 0.080 g L^−1^ d^−1^ dw (Table 1). It was also shown that these strains were able to produce and accumulate carotenoids in response to nutritional and highlight stresses (>750 µmol photons m^−2^ s^−1^) (Figure 4). Chromatographic and spectrometric analyses highlighted the presence of canthaxanthin and different forms of astaxanthin in all biomasses of these stressed strains (Figure 5, Figure 6, Figure 7, Figure 8 and Figure 9; Table 2). However, the strain CBR11 presented some particularities concerning its carotenoid profile, probably reflecting the morphological and genetic differences previously shown in another study [16]. In this strain, canthaxanthin and astaxanthin contents could reach 1.15 and 0.67 mg g^−1^ dw, respectively, corresponding to productivities of around 0.06 and 0.04 mg L^−1^ d^−1^ dw, respectively (Table 3).

### 3.1. Dysmorphococcus Strains Cultivation

To evaluate the growth potential of the isolated *Dysmorphococcus* strains in culture under laboratory conditions, cells were grown in batch mode with different nutritive media at different scales. The best biomass productions were obtained for the CBR1 and CBR11 strains which developed well in the 3N-BBM medium. Figure 3 presents the growth kinetics monitored for the CBR11 strain in a 2 L glass reactor and a 3 L benchtop photobioreactor (Figure 3a and Figure 3b, respectively), as well as for the CBR1 strain in a 3 L benchtop photobioreactor (Figure 3c). The growth kinetics are compared in Figure 3d, in which cell densities are expressed in a logarithmic scale.

Based on these curves, growth parameters were calculated and are presented in Table 1. Concerning CBR11, a higher *µ_max_* and *µ_mean_* (0.32 d^−1^ and 0.18 d^−1^, respectively) were obtained for the culture in a 3 L benchtop photobioreactor compared to that of the culture in a 2 L glass reactor (0.26 d^−1^ and 0.15 d^−1^, respectively). However, the maximum cell densities were not statistically different (approximately 1800 × 10^3^ cells mL^−1^), suggesting that irradiances around 200–250 µmol photons m^−2^ s^−1^ were sufficient for an admissible vegetative growth.

After 77 days, the biomass concentration for the strain CBR11 in the benchtop photobioreactor reached more than 4 g L^−1^ dw corresponding to a biomass productivity of approximately 0.055 g L^−1^ d^−1^ dw. At this time, after a period of nutritional and highlight stresses, the culture appeared bright orange, indicating an accumulation of carotenoids (Figure 4).

With respect to the CBR1 strain cultivated in a 3 L photobioreactor, the results were comparable to those obtained for CBR11 with respect to *µ_max_* (0.33 d^−1^) and to the maximum cell density (more than 1900 × 10^3^ cells mL^−1^), but the duration of the vegetative growth was significantly longer corresponding to a lower *µ_mean_* (0.13 d^−1^) (Table 1). However, after 68 days, a higher biomass concentration of almost 5.5 g L^−1^ dw was obtained for the strain CBR1 corresponding to a biomass productivity of 0.08 g L^−1^ d^−1^ dw (Table 1).

### 3.2. Chromatographic and Spectrometric Analyses

#### 3.2.1. Comparative Qualitative Analyses of the Pigment Extracts Obtained from the Isolated *Dysmorphococcus* Strains

To assess whether secondary carotenoid profiles of the isolated *Dysmorphococcus* strains reflected the genetic and morphological differences highlighted in our previous study [16], pigment extracts obtained from biomasses after carotenogenesis induction by application of nutritive and light stresses were qualitatively analyzed by HPTLC and HPLC in the reverse phase (Figure 5 and Figure 6, respectively).

HPTLC chromatograms and densitograms (Figure 5a,c, respectively) showed that pigment extracts from the *Dysmorphococcus* strains overall presented similar carotenoid profiles (tracks 3–10) characterized by two intense bands at retention factors (R_F_) of 0.49 and 0.58, which could correspond to the main astaxanthin ester found in the reference extract from *H. lacustris* (i.e., the more intense red band in track 2) and canthaxanthin (i.e., the corresponding peak in the carotenoid standard mix solution in track 1), respectively. These chromatograms also presented a band at the same R_F_ as free astaxanthin (R_F_ = 0.67) and at least four bands (R_F_ = 0.32, 0.38, 0.45 and 0.53), more or less intense, potentially corresponding to other astaxanthin esters. Moreover, two bands at R_F_ = 0.42 and R_F_ = 0.62 could be attributed to the primary carotenoids β, β-carotene and lutein, respectively.

However, some differences were noticeable. Indeed, the pigment extracts from the strain CBR7 (track 8) presented a carotenoid profile in which primary carotenoids (β, β-carotene and lutein) are still relatively present, unlike other extracts whose profiles seemed to be dominated by secondary carotenoids (astaxanthin and canthaxanthin). This could be explained by a difference in the physiological development stage and/or by some genetic characteristics which limited the production of secondary carotenoids in this strain. On the contrary, the pigment extract from the strain CBR11 (Figure 5a,c, track 9) presented the most similar carotenoid profile to that of the reference extract of astaxanthin esters from *H. lacustris*, except regarding the presence of canthaxanthin. Indeed, for this extract, at least eight bands could be attributable to astaxanthin esters (R_F_ = 0.32, 0.35, 0.38, 0.42, 0.45, 0.49, 0.53 and 0.56) with higher relative intensities than for the extracts of the other strains. In addition, the illumination of the HPTLC plate at 366 nm (Figure 5b) revealed differences in the chlorophyll profile for the strain CBR5 (track 7) and the strain CBR11 (track 9) compared to the other strains.

The comparative qualitative HPLC analysis of these extracts (Figure 6) confirmed the previous results and highlighted the predominance of canthaxanthin in all the isolated *Dysmorphococcus* strains (peak 11 with a retention time (Rt) at 16.08 min and with a wavelength of maximum absorbance (λ_max_) at 473 nm). The HPLC chromatograms also showed the higher relative abundance of the potential astaxanthin esters among secondary carotenoids in the extracts of CBR11 (Figure 6, chromatogram g) compared to the other extracts.

#### 3.2.2. Qualitative and Quantitative Analyses of the Pigment Extract Obtained from the Isolated *Dysmorphococcus* Strain CBR11

Because of its physiological, morphological, genetic and biochemical particularities compared to the other isolated *Dysmorphococcus* strains, CBR11 was selected for more in-depth qualitative and quantitative chromatographic and spectrometric analyses at different stages of its life cycle (Figure 7, Figure 8 and Figure 9; Table 2).

The HPTLC chromatograms and densitograms of the extracts obtained from the green biomass of the strain CBR11 (Figure 7, track 3) showed at least seven bands at R_F_ = 0.39, 0.43, 0.57, 0.62, 0.68, 0.81 and 0.85 with, for each, two absorption maxima at 410–425 nm and 640–668 nm. These bands could be attributed to chlorophylls and/or their derivatives and precursors. The chromatograms also presented two bands (R_F_ = 0.43; λ_max_ at 456 nm and R_F_ = 0.62; λ_max_ at 450 and 474 nm) which could correspond to β, β-carotene and lutein, respectively. In addition, an undetermined carotenoid was detected at R_F_ = 0.73 and presented three absorption peaks at wavelengths of 405, 427 and 455 nm.

The chromatograms and densitogram of the extracts from the orange biomass (Figure 7, track 4) were similar to that presented in Figure 5 (track 9). More precisely, they also showed at least eight bands (R_F_ = 0.35, 0.38, 0.41, 0.43, 0.47, 0.50, 0.54 and 0.56) with absorption maxima comprised between 485 and 496 nm, probably corresponding to astaxanthin esters, as well as a band absorbing at 486 nm with the same R_F_ than the free astaxanthin standard (R_F_ = 0.67) (Figure 7, track 1). Moreover, a relatively intense band at R_F_ = 0.59 (λ_max_ at 486 nm) and a light band at R_F_ = 0.63 (λ_max_ at 451 nm and 478 nm) could be attributable to canthaxanthin and lutein, respectively. Chlorophylls were still present in these extracts with three detectable bands at R_F_ = 0.39, 0.57 and 0.62, each presenting two absorption maxima at 415–426 nm and 653–668 nm. After saponification of these extracts, the chromatograms and the densitograms presented only two bands corresponding to canthaxanthin and lutein (R_F_ = 0.59; λ_max_ at 486 nm and R_F_ = 0.63; λ_max_ at 456 nm and 481 nm, respectively) and one band representing free astaxanthin (R_F_ = 0.66; λ_max_ at 490 nm), which became significantly more intense than before saponification (Figure 7a,c, track 5). Additionally, at least two bands were detectable under illumination of the HPTLC plate at 366 nm (R_F_ = 0.77 and 0.81; λ_max_ at 406–407 nm and 644 nm) and probably corresponded to chlorophyll derivatives (Figure 7b, track 5).

The HPLC analyses confirmed the presence of chlorophylls or their derivatives in the extracts obtained from the green biomass of the strain CBR11 (Figure 8a,d; Table 2). Indeed, at least six peaks, each presenting two absorption maxima at 407–467 nm and 650–667 nm, were detected in these extracts at Rt = 10.96 (peak 1), 12.25 (peak 3), 15.18 (peak 9), 16.07 (peak 10), 26.75 (peak 25) and 28.18 min (peak 27). A peak at Rt = 13.40 min with λ_max_ at 442 nm and 470 nm (peak 5) could be attributed to lutein, while at least four small peaks eluted between 20.30 and 23.85 min and absorbing at 479 nm (peaks 17, 18, 20 and 23) were probably traces of astaxanthin monoesters. These peaks represented approximately 2.3% of the total peak area of the pigment detected in the extract at 480 nm. Surprisingly, β, β-carotene, yet visible in HPTLC, was not detected in these extracts—it was probably hidden by a chlorophyll (peak 25).

The HPLC chromatograms of the pigment extracts obtained from the orange biomass of the strain CBR11 (Figure 8b,e; Table 2) presented a majority peak at Rt = 16.08 with a min absorbance at 471 nm, which could be attributed to canthaxanthin (peak 11). This peak represented around 16% of the total peak area of the pigments detected at 480 nm in the extract. Moreover, at least twenty peaks absorbing between 465 and 482 nm and representing more than 64% of the total peak area of the detected pigments could correspond to different forms of astaxanthin. These peaks included a potential peak of free astaxanthin (peak 4, Rt = 12.36 min) accounting for approximatively 6% of the total amount of astaxanthin, eight peaks of astaxanthin monoesters (peaks 17, 18, 20–24 and 26; Rt from 20.30 to 27.85 min) representing around 73%, and eleven peaks of astaxanthin diesters (peaks 28–38; Rt from 30.26 to 35.24 min) for 21%. The peak attributed to lutein (peak 5) was still detected in these extracts. In addition, nine peaks presenting absorption maxima between 453 and 468 nm (peak 2 at Rt = 11.78 min, peaks 6 and 7 at Rt from 13.92 to 14.44 min, peaks 12–16 at Rt from 17.26 to 19.91 min and peak 19 at Rt = 21.41 min) could be attributed to carotenoids that remained undetermined. LC-MS analyses confirmed the presence of free astaxanthin (peak 4) with a mass/charge ratio (*m*/*z*) of the corresponding molecular cation ([M + H]^+^) of 597.0 and canthaxanthin (peak 11; *m*/*z* [M + H]^+^ = 565.0) (Figure 9a,b; Table 2). Moreover, four astaxanthin monoesters could also be analyzed by LC-MS and the results showed that they were composed of linolenic (C18:3) (peak 18; *m*/*z* [M + H]^+^ = 857.5), linoleic (C18:2) (peak 20; *m*/*z* [M + H]^+^ = 859.6), oleic (C18:1) (peak 22; *m*/*z* [M + H]^+^ = 861.6) and palmitic (C16:0) (peak 23; *m*/*z* [M + H]^+^ = 835.5) acids (Figure 9c; Table 2). The main astaxanthin monoester (peak 20) which represented approximately 18% of the total amount of astaxanthin was, therefore, composed of C18:2. Other astaxanthin esters could not be analyzed by LC-MS because they were present in too low of an amount in the samples. However, considering the similarities with respect to Rf and λ_max_ between some peaks of these extracts and those of the reference extract from *H. lacustris,* as well as the results obtained by LC-MS for the reference extract, it could be suggested that peak 26 could correspond to astaxanthin esterified with stearic acid (C18:0) and that peaks 31–35 were astaxanthin diesters (Table 2). Based on this hypothesis, peak 31 would be composed of C18:1/C18:3 or C18:2/C18:2, peak 32 of C18:1/C18:2 or C18:0/C18:3, peak 33 of C16:0/C18:3 or C16:1/C18:2, peak 34 of C18:1/C18:1 or C18:0/C18:2 and peak 35 of C16:0/C18:1 or C16:1/C18:0.

After saponification, the pigment extracts still contained mostly canthaxanthin (peak 11), which accounted for almost 39% of the total peak area of the detected pigments at 480 nm, but also lutein (peak 5) and some of the undetermined carotenoids (Figure 8c,f). However, no peaks corresponding to astaxanthin esters were detected, while the area of the peak attributed to free astaxanthin significantly increased, as expected, representing more than 23% of the total peak area of the detected pigments at 480 nm.

To evaluate the productivities of the production of valuable carotenoids by *Dysmorphococcus* strain CBR11, canthaxanthin and free astaxanthin were quantified by HPLC after saponification of the extracts on the basis of the respective calibration curves, which are presented in Supplementary Figure S1. The results obtained for a biomass produced in a 3 L photobioreactor are presented in Table 3 and showed that the content of canthaxanthin and free astaxanthin equivalent reached 1.15 mg g^−1^ dry weight (dw) and 0.67 mg g^−1^ dw, respectively. On the basis of the biomass productivities calculated for this culture (Table 1), the potential productivities of canthaxanthin and astaxanthin were approximately of 0.06 mg L^−1^ d^−1^ dw and 0.04 mg L^−1^ d^−1^ dw, respectively.

## 4. Discussion

In this study, eight newly isolated *Dysmorphococcus* strains from Reunion Island could be cultivated in laboratory conditions in 250 mL glass flasks, and it was shown that they were able to produce and accumulate carotenoids after exposure to stresses, whether nutritional or high levels of light. As the strains CBR1 and CBR11 exhibited relatively better growth at a small scale compared to the other strains, their cultures were chosen to be scaled up to a volume of 3 L in benchtop photobioreactors. The preliminary cultures of these strains at this scale were carried out in batch mode using 3N-BBM and under growing irradiance depending on cell densities, under conditions that remained unoptimized (Figure 3 and Table 1). Despite this, the maximum and the mean growth rates obtained for strain CBR1 were 0.33 d^−1^ and 0.13 d^−1^, respectively, and 5.5 g L^−1^ of dry biomass enriched in carotenoids could be harvested after 68 days, corresponding to a biomass productivity of 0.08 g L^−1^ d^−1^ dw. The maximum growth rate obtained for the culture of strain CBR11 was similar (0.32 d^−1^), while the mean growth rate was significantly higher (0.18 d^−1^). However, after 77 days of culture, 4.25 g L^−1^ of dry biomass enriched in carotenoids could be harvested, corresponding to a biomass productivity of 0.055 g L^−1^ d^−1^ dw less than that obtained for CBR1. On the basis of these preliminary assays, it was difficult to conclude whether the differences in growth kinetics and biomass productivities reflected strain particularities or were due to differences in growing conditions, particularly with regard to light regime. Even if maximal cell densities were not significantly different between the cultures of CBR11 in the 2 L bioreactor and in the 3 L benchtop photobioreactor, the growth was found to be faster in the photobioreactor probably because it allows for better light availability due to its shorter optical length, more efficient agitation, and better illumination in a wider range of irradiances than the 2 L bioreactor. In any case, biomass productivities for these two strains could certainly be improved by optimizing the process, particularly with regard to the availability of nutrients and light, the regulation of environmental conditions such as pH and temperature, and the cultivation regime.

The chromatographic analyses carried out on pigment extracts from the biomass of the eight isolated *Dysmorphococcus* strains showed that they respond to an exposure to stresses, whether nutritional or high levels of light, through the production and accumulation of secondary carotenoids, especially canthaxanthin and astaxanthin (Figure 5 and Figure 6). If these chromatographic analyses showed similarities between the carotenoid profiles of these strains, they also showed significant differences, particularly with regard to strain CBR11. These biochemical differences potentially reflected, at least in part, the genetic and morphological differences previously highlighted compared with the other locally isolated *Dysmorphococcus* strains [16]. This result provided additional evidence supporting that the CBR11 strain could belong to a particular species of *Dysmorphococcus*. In this strain, qualitative chromatographic and mass spectrometric analyses showed that the main secondary carotenoids were canthaxanthin and astaxanthin and that astaxanthin was mainly found esterified with one fatty acid, mostly linoleic acid, but also in the form of diesters and, to a lesser extent, as free astaxanthin (Figure 7, Figure 8 and Figure 9; Table 2).

Astaxanthin is a ketocarotenoid which can be valuable in the fields of human nutrition, health and cosmetics, particularly for its numerous biological activities including its remarkable antioxidant properties [25], as well as feed and aquaculture for enhanced pigmentation, quality and productivities [26]. For more details, the biological and biochemical properties of astaxanthin were reviewed in a previous study, along with its commercial production through the culture of the freshwater green microalgae *H. lacustris,* which is considered to be one of the best sources of natural astaxanthin [27], but the reader can also refer to other reviews [28,29]. As mentioned above, the potential production of natural astaxanthin by *Dysmorphococcus* species was previously mentioned in a study dedicated to a Himalayan strain of *D. globosus* [15], but, to our knowledge, none have suggested their ability to produce and accumulate canthaxanthin.

**Figure 10:**
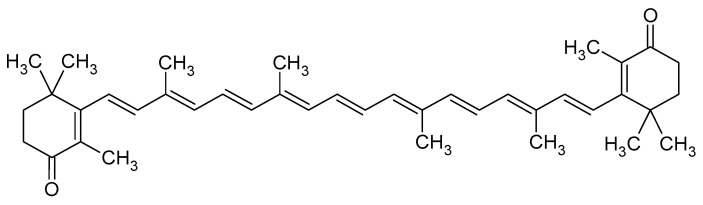
Molecular structure of all-*trans*-canthaxanthin [31].

Canthaxanthin, or β,β-carotene-4,4′-dione, is a red-orange xanthophyll carotenoid of molecular formula C_40_H_52_O_4_ and a molecular mass of 564.8 g mol^−1^ [30]. As astaxanthin, this tetraterpene is composed of two β-ionone rings joined by a nonpolar central polyene chain and contains 13 conjugated double-bonds which constitute the chromophore of the molecule. However, each cycle contains only one keto moiety (Figure 10); therefore, canthaxanthin has no chiral centers [31] and cannot be esterified with fatty acids, in contrast to astaxanthin.

Canthaxanthin was first isolated from the mushroom *Cantharellus cinnabarinus* and is widely distributed in other fungi, but also in bacteria, algae, crustaceans and the feathers of birds [31]. Table 4 summarizes the canthaxanthin yields of some microorganisms found in the literature. Provided by the diet, canthaxanthin is involved in the coloration of flamingo feathers, koi carp skin, salmon and trout flesh and egg yolk [30]. Moreover, some in vitro and in vivo studies have highlighted its high antioxidant and free radical scavenging properties, as well as its immunomodulatory and anticancer activities [32]. Thus, natural or synthetic canthaxanthin is mainly valuable in the fields of feed and aquaculture but also for human applications such as cosmetics, for example as a skin-tanning agent [30], dietary supplements, food additives (E 161 g) [2] and pharmaceutical products. According to some economic analysts, the canthaxanthin market size was valued at USD 135 million in 2023 and could reach USD 444 million in 2032 with a CAGR of 2.6% [33].

In numerous microalgae, canthaxanthin was found in combination with astaxanthin, for example for *H. lacustris* [46], *Chlorella zofingiensis* [35,47], *C. vulgaris* [48], *Chlorococcum* sp. [36], *Coelastrella striolata* [37] and *Scenedesmus quadricauda* [39]. In fact, canthaxanthin is one of the precursors of astaxanthin in its biosynthetic pathway through its action in the cytoplasm of the enzyme *β*-carotene hydroxylase (CrtR-b or CrtZ) which hydroxylates canthaxanthin to form adonirubin, which is then hydroxylated in astaxanthin [49,50]. However, canthaxanthin appeared to be converted into astaxanthin only in reduced amounts in the locally isolated *Dysmorphococcus* strains.

The quantitative analyses by HPLC showed that the strain CBR11 can accumulate canthaxanthin and astaxanthin with contents that reach almost 1.2 mg g^−1^ dw and 0.7 mg g^−1^ dw, respectively, corresponding to the respective productivities of 0.06 and 0.04 mg L^−1^ d^−1^ dw (Table 3). With respect to the production of natural astaxanthin, the culture of the *Dysmorphococcus* strain CBR11 did not appear competitive compared to that of the microalgae *H. lacustris* [27], or even the isolated *Haematococcus* strains from Reunion Island studied in our previous work [51]. To be updated after the review process). Indeed, it was previously shown that *H. lacustris*, which can accumulate astaxanthin representing up to 4% *w*/*w* of dry biomass [52], could be cultivated to produce astaxanthin, with the astaxanthin productivity reaching 21 mg L^−1^ d^−1^ dw [53], which is considerably higher than our results. On the other hand, the canthaxanthin concentration in the cultures of CBR11 (4.89 mg L^−1^) was comparable to that of some microalgae investigated for their production of this carotenoid, such as *B. braunii*, *C. zofingiensis* or *D. dissociatus* (Table 4). However, compared to *C. striolata,* which presented a much greater canthaxanthin content (47.5 mg g^−1^ dw), or *Chlorococcum* sp., whose biomass concentration in the culture could reach 18 g L^−1^ dw with a *µ_max_* of 0.8 day^−1^ [36], the locally isolated *Dysmorphococcus* strain CBR11 did not appear to be the best candidate for current commercial production of natural canthaxanthin. Even if these results seem relatively low compared with those obtained with other microalgae, they are encouraging for a recently isolated wild strain grown under non-optimal conditions. Astaxanthin and/or canthaxanthin productivities could certainly be improved by optimizing the cultivation process, particularly to enhance vegetative growth and carotenogenesis and consequently to reduce cultivation duration. This is why our future research will focus on more in-depth studies with regard to the availability of nutrients and light, the regulation of parameters such as pH and temperature, the choice of a suitable cultivation regime and the application of combined stresses inducing carotenogenesis. Moreover, research could be extended to the other isolated *Dysmorphococcus* strains, which could prove potentially more productive in terms of biomass and/or secondary carotenoids. On the other hand, while some microalgae are known to produce a variety of toxins that are harmful or lethal to humans, nothing has yet been demonstrated for *Dysmorphococcus*. Thus, the question of the ability of this strain to produce toxins harmful to humans remains open. Before considering industrial production of astaxanthin and canthaxanthin by these newly isolated *Dysmorphococcus* strains for human applications such as nutrition or food, it will therefore be necessary to carry out a toxicity study, for example using the zebrafish model, of the biomasses and colored extracts. These results will be the subject of a future communication on upscaling and large-scale production of astaxanthin and canthaxanthin from these strains.

## 5. Conclusions

As a conclusion, this study demonstrated that the *Dysmorphococcus* strains isolated from the biodiversity of Reunion Island were able to produce and accumulate secondary carotenoids, such as canthaxanthin and astaxanthin, in response to environmental stresses. Among them, strain CBR11 presented a particular carotenoid profile compared to the other *Dysmorphococcus* strains, which could reflect the morphological and genetic differences previously highlighted. This strain was able to grow relatively satisfactorily in a 3 L benchtop photobioreactor using the 3N-BBM medium in batch mode, with a biomass productivity reaching more than 0.05 g L^−1^ d^−1^ dw based on the total cultivation time. After exposure to environmental stresses, the biomass could contain almost 1.2 mg g^−1^ dw and 0.7 mg g^−1^ dw of canthaxanthin and astaxanthin, respectively, with respective productivities of around 0.06 and 0.04 mg L^−1^ d^−1^ dw. These results were significantly lower compared to some microalgae that are potentially more productive and further optimizations are necessary to make this strain more competitive for the commercial production of natural canthaxanthin and astaxanthin.

## Figures and Tables

**Table 1:** Growth parameters of the *Dysmorphococcus* strains CBR11 and CBR1 cultivated in batch mode with the 3N-BBM medium under increasing PPFD.

Parameter	CBR11		CBR1
	2 L Reactor	3 L Photobioreactor	3 L Photobioreactor
*µ_max_* (d^−1^)	0.26	0.32	0.33
*td_min_* (d)	2.69	2.17	2.10
*Cd_max_* (×10^3^ cells mL^−1^)	1825.00 ± 260.69 ^a^	1742.50 ± 104.04 ^a^	1957.50 ± 177.65 ^a^
Time for Cd_max_ (d)	25	19	68
*µ_mean_* (d^−1^)	0.15 *	0.18 **	0.13 ***
*td_mean_* (d)	4.49 *	3.89 **	5.34 ***
Dry biomass (g L^−1^)	Nd	4.25	5.47
Cultivation time (d)	52	77	68
Biomass productivity (g L^−1^ d^−1^)	Nd	0.055	0.080

Data presented as mean ± standard deviation (*n* = 4). Letter a in the same line indicates that there were no significant difference among results using Tukey’s test (*p* < 0.05). * Calculated from day 11 to day 21; ** calculated from day 4 to day 14; *** calculated from day 0 to day 20. Nd: not determined.

**Table 2:** Attempts to identify the detected pigments extracted from the *Dysmorphococcus* strain CBR11 using chromatographic and spectrometric data in comparison with an *H. lacustris* reference extract.

Peak nb	Retention Time (min)	Compound	*H. lacustris* Reference Extract	CBR11 Green		CBR11 Orange		CBR11 Orange Sapo		*m*/*z* [M + H]^+^
			λmax (nm)	λmax (nm)	Relative Peak Area ^a^	λmax (nm)	Relative Peak Area ^a^	λmax (nm)	Relative Peak Area ^a^	
1	10.96	Chl	-	**467**, 650	49.15	nd	0.96	nd	1.06	-
2	11.78	UC	-	-	-	nd	1.08	402, **468**	7.47	-
3	12.25	Chl	-	**420**, 661	6.14	-	-	-	-	-
4	12.36	Free Ax	479	-	-	471	3.69	474	23.45	597.0
5	13.40	Lutein	442, **469**	**442**, 470	21.65	**439**, 469	1.42	**443**, 469	3.93	-
6	13.92	UC	469	-	-	363, **462**	2.36	364, **466**	5.95	-
7	14.44	UC	478	-	-	362, **468**	1.85	363, **472**	8.27	-
8	14.91	UC	-	-	-	-	-	360, 471, **479**	3.53	-
9	15.18	Chl	-	**430**, 664	2.11	-	-	-	-	-
10	16.07	Chl	-	**433**, 664	1.97	-	-	-	-	-
11	16.08	Canthaxanthin	476	-	-	472	16.29	473	38.80	565.0
12	17.26	UC	475	-	-	461	0.96	467	2.39	-
13	17.67	UC	**458**, 468	-	-	457, **465**	0.51	449, **469**	1.80	-
14	18.89	UC	468	-	-	461	1.37	-	-	-
15	19.21	UC	471	-	-	463	1.72	-	-	-
16	19.91	UC	468	-	-	464	1.26	-	-	-
17	20.30	Ax monoester	469	479	1.18	468	6.85	-	-	-
18	20.71	Ax-C18:3	475	479	0.46	473	6.82	-	-	857.5
19	21.41	UC	-	-	-	453, **467**	2.16	-	-	-
20	21.98	Ax-C18:2	474	479	0.66	472	11.13	-	-	859.6
21	22.76	Ax monoester	471	-	-	465	1.21	-	-	-
22	23.48	Ax-C18:1	469	-	-	468	2.13	-	-	861.6
23	23.85	Ax-C16:0	475	479	0.66	476	7.14	-	-	835.5
24	25.09	Ax monoester	472	-	-	470, **480**	5.47	-	-	-
25	26.75	Chl	-	**410**, 666	0.95	-	-	-	-	-
26	27.85	Ax-C18:0	477	-	-	472	6.00	-	-	863.7 **
27	28.18	Chl	-	**407**, 665	2.57	-	-	-	-	-
28	30.26	Ax diester	473	-	-	479	0.59	-	-	-
29	31.10	Ax diester	476	-	-	478	0.84	-	-	-
30	31.29	Ax diester	474	-	-	480	0.34	-	-	-
31	31.65	Ax-C18:1/C18:3 *	475	-	-	479	1.28	-	-	1122.0 **
32	31.84	Ax-C18:1/C18:2 *	475	-	-	478	1.34	-	-	1124.0 **
33	32.41	Ax-C16:0/C18:3 *	475	-	-	477	1.87	-	-	1096.6 **
34	32.80	Ax-C18:1/C18:1 *	475	-	-	480	1.28	-	-	1126.0 **
35	33.44	Ax-C16:0/C18:1 *	476	-	-	477	1.63	-	-	1100.0 **
36	34.20	Ax diester	475	-	-	472, **482**	1.14	-	-	-
37	34.62	Ax diester	469	-	-	474	2.17	-	-	-
38	35.24	Ax diester	473	-	-	474	1.19	-	-	-
Canthaxanthin ^a,c^					0.0		16.3		38.8	
Total astaxanthin ^a,c^					2.3		64.1		23.5	
Free astaxanthin ^b,c^					0.0		5.8		100.0	
Astaxanthin monoesters ^b,c^					100.0		72.9		0.0	
Astaxanthin diesters ^b,c^					0.0		21.3		0.0	

Chl: chlorophyll or derivative; UC: undetermined carotenoid; Ax: astaxanthin; Nd: not determined. * Possible fatty acid combination; ** results obtained for the reference extract of *H. lacustris*; ^a^ % of total peak area of the extract considering all pigments detected at 480 nm; ^b^ % of total astaxanthin; ^c^ ratios based on the relative peak areas at 480 nm. Absorbance maxima are indicated in bold. *m*/*z*: ratio of an ion’s mass (*m*) to its formal charge (*z*); [M + H]^+^: molecular cation.

**Table 3:** Canthaxanthin and astaxanthin contents measured by HPLC in the orange biomasses of the strain CBR11 cultivated in a 3 L benchtop photobioreactor after saponification of the extracts.

Parameter	CBR11 FMT
Canthaxanthin content (mg g^−1^ dw)	1.15
Astaxanthin content (mg g^−1^ dw)	0.67
Canthaxanthin concentration (mg L^−1^ dw) ^1^	4.89
Astaxanthin concentration (mg L^−1^ dw) ^1^	2.85
Canthaxanthin productivity (mg L^−1^ d^−1^ dw) ^1^	0.06
Astaxanthin productivity (mg L^−1^ d^−1^ dw) ^1^	0.04

^1^ Calculated from the results presented in Table 1.

**Table 4:** Summary of canthaxanthin yields obtained by the cultivation of different microorganisms found in the literature compared with the results of this study.

Type	Genus/Species	Biomass Concentration (g L^−1^ dw)	Canthaxanthin Content (mg g^−1^ dw)	Canthaxanthin Concentration (mg L^−1^ dw)	TC Content (% *w/w*)	Reference
Yeast	*Saccharomyces cerevisiae* (genetically modified)	-	9.63 ^a^	-	-	[32]
Microalgae	*Botryococcus braunii*	2.3	1.7 ^a^	3.9 ^a^	0.37	[34]
	*Chlorella zofingiensis*	0.57	8.5	4.85 ^a^	1.17 **	[35]
	*Chlorococcum* sp.	18.0	1.95	35.1 ^a^	0.56 *	[36]
	*Coelastrella striolata*	2.5–3	47.5	118.8–142.5 ^a^	5.6 **	[37]
	*Dactylococcus dissociatus*	2.25	1.74 ^a^	3.92	-	[38]
	*Scenedesmus quadricauda*	3.7	4.0	14.8 ^a^	9.8	[39]
	*Dysmorphococcus* strain CBR11	4.25	1.15	4.89	-	This study
Bacteria	*Bradyrhizobium* sp.	0.58 ^a^	1.34	0.78	0.16	[40]
	*Brevibacterium* sp.	8.2–12.6	0.74–0.88	7.2–9.3	-	[41]
	*Dietzia maris*	-	-	0.97	-	[42]
	*Dietzia natronolimnaea* (mutant)	9.89	1.42 ^a^	6.98	-	[43]
	*Gordonia jacobaea* (mutant)	-	0.97	-	-	[44]
Archaea	*Haloferax alexandrinus*	3.12	2.16	6.74 ^a^	0.63	[45]

TC: Total carotenoids. ^a^ Calculated from the results of the study; * Total ketocarotenoids; ** Total secondary carotenoids.

## Data Availability

The original contributions presented in the study are included in the article; further inquiries can be directed to the corresponding authors.

## Supplementary Materials

The following supporting information can be downloaded at: https://www.mdpi.com/article/10.3390/foods13233922/s1. Figure S1: Calibration curves obtained by HPLC at 480 nm: (a) for canthaxanthin; (b) for free astaxanthin. Markers represent the average of analytical standard injections (*n* = 3–4).
