# Supplementary material for: Study on Newly Isolated Dysmorphococcus Strains from Reunion Island as Potential Sources of High-Value Carotenoids
Source: Foods. 2024 Dec 4;13(23):3922. doi: 10.3390/foods13233922 (PMC11641088; doi:10.3390/foods13233922)
Supplement: Supplementary file 1 [file foods-13-03922-s001.zip › foods-3295237-supplementary.pdf]

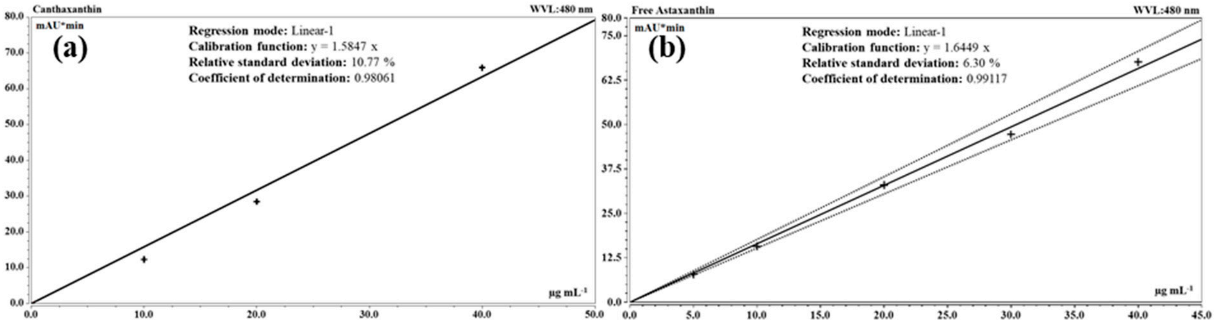

Figure S1. Calibration curves obtained by HPLC at 480 nm: (a) for canthaxanthin; (b) for free astaxanthin. Markers represent the average of analytical standard injections (n = 3–4).
